# Effect of Particle Size on Physicochemical Properties and *in vitro* Hypoglycemic Ability of Insoluble Dietary Fiber From Corn Bran

**DOI:** 10.3389/fnut.2022.951821

**Published:** 2022-07-15

**Authors:** Caixia Jiang, Rui Wang, Xiaolan Liu, Juntong Wang, Xiqun Zheng, Feng Zuo

**Affiliations:** ^1^National Coarse Cereals Engineering Research Center, Heilongjiang Bayi Agricultural University, Daqing, China; ^2^College of Food Science, Heilongjiang Bayi Agricultural University, Daqing, China; ^3^College of Food and Bioengineering, Qiqihar University, Qiqihar, China; ^4^Heilongjiang Key Laboratory of Corn Deep Processing Theory and Technology, Qiqihar, China

**Keywords:** insoluble dietary fiber, particle size, physicochemical properties, structural characteristics, hypoglycemic ability

## Abstract

This study was designed for determining the effect of particle size on the functional properties of corn bran insoluble dietary fiber (IDF). Results showed that some physicochemical properties were improved with the decrease in particle size. The structure of the IDF was observed by the scanning electron microscope (SEM), X-ray diffraction (XRD), and Fourier transform infrared spectroscopy (FT-IR). The surface was found wrinkled and sparse, the particle size was smaller, the crystallinity of IDF had increased slightly, and more -OH and C-O groups were exposed. Moreover, the corn bran IDF with a smaller particle size had a better hypoglycemic effect *in vitro*, and the inhibitory activity of α-glucosidase and α-amylase were also increased significantly with the decrease in particle size (*p* < 0.05). When the IDF was 300 mesh, the inhibitory rate of α-glucosidase was 61.34 ± 1.12%, and the inhibitory rate of α-amylase was 17.58 ± 0.33%. It had increased by 25.54 and 106.83%, respectively compared to the control treatment (CK) group. In addition, correlation analysis found that the particle size was highly negatively correlated with some functional properties of IDF (*p* < 0.05), and the content of cellulose was positively correlated with the functional properties of IDF except WHC (*p* < 0.05). To sum up, reducing particle size was suitable for the development of high value-added IDF products. This study also revealed the potential value of corn bran IDF and provided a new idea for the diversified application of IDF.

## Introduction

Dietary Fiber (DF) is a polysaccharide carbohydrate with low nutritional value, but it has a variety of benefits for human health and reduces the risk of diabetes (1, 2). It could be utilized in the formulation of new food or health products (3). DF can be divided into soluble dietary fiber (SDF) and IDF. Some studies have confirmed that IDF can effectively improve intestinal function, thereby regulating lipid metabolism (4). Furthermore, functional properties, for example, the cholesterol adsorption capacity (CAC) and the oil holding capacity (OHC), are mainly due to IDF than SDF (5). To achieve optimal health benefits and function, it is recommended to include 25–50 % of IDF in the DF (6).

Corn is the world's largest food crop in terms of total output and has immense economic and social value. Corn produces many by-products during its processing, such as corn cob, corn bran, corn gluten meal, and corn fiber (7, 8). In general, corn bran is about 14–20% of the total corn. Due to its properties such as rough taste, poor water solubility, etc., the wide application of corn bran is limited (9). However, it has great significance for in-depth research given that the main part of corn bran is a type of hemicellulose (10). Also, different processing methods of corn bran can be used to obtain oligosaccharides and polysaccharides with different degrees of polymerization.

Ultrafine grinding has always been considered an efficient and green food processing technology (11, 12). The particles of raw materials are processed into micro or even nanometers through air separation, heavy pressure grinding, shearing, and subject to impact or dynamic flow to overcome the cohesive force of solids (13) until the powder with uniform particle size is obtained. This process not only improved the physical, chemical, functional, and edible properties of the raw materials, but also promoted the release and absorption of nutrients and bioactive components in the raw materials, which realized the full-price utilization of the raw materials and expanded its application in food resources (14). Ultrafine grinding technology has been widely used in the processing of wheat, corn, beans, and a few Chinese herbal medicines. Among DFs, potato peel total dietary fiber, fruit pomace dietary fiber, and bamboo shoot have a wide range of applications (15, 16). Powders with smaller particle sizes have better fluidity and hydration, lower interfacial tension, and better flavor and texture (17). Furthermore, a higher degree of micronization can promote changes in some groups, for instance, in chemical bonds in DFs where some fiber components were transformed from being insoluble to soluble (18). However, not all materials are suitable for ultrafine grinding; also, not all finer powders are better. At present, there are few reports on IDF being subject to ultrafine pulverization technology, especially, when it is a by-product of agricultural products. At the same time, the change in the physicochemical properties and microstructure of IDF from corn bran is not clear.

In this study, the IDF from corn bran was obtained by using enzymatic hydrolysis. The ultrafine corn bran IDF powder was prepared using an airflow pulverizer and sieved with different mesh sieves to obtain corn bran IDF in different particle sizes. This study aimed to explore the effect of particle size on the IDF's functions. It provides technical support for corn bran IDF as a functional ingredient for hyperglycemia and plays an active role in improving the added value of corn bran.

## Materials and Methods

### Materials and Chemicals

Corn bran was purchased in 2021 from Beijing Jingliang Co., Ltd. (Suihua, China). and stored at 4°C for use. Alkali protease (2 × 10^5^ u/g), saccharifying enzyme (1.5 × 10^5^ u/g), and amylase (2 × 10^5^ u/g) were purchased from Novozymes Biotechnology Co., Ltd. (Shanghai, China). Total Starch Assay Kit and Glucose Assay (ab65333) were purchased from Abcam Shanghai Trading Co., Ltd. (Shanghai, China). α-glucosidase (50 U/mg), and 4-nitrophenyl-α-D-glucopyranoside (PNPG) were purchased from Yuanye Bio-Technology Co., Ltd. (Shanghai, China). Cellulose(CLL)Content Assay Kit (AKSU007C), Themicellulose Content Assay Kit (AKSU008C), and Lignin Content Assay Kit (AKSU010U) were purchased from Beijing Boxbio Co., Ltd. (Beijing, China).

### Preparation of Corn Bran IDF

The preparation of corn bran IDF was referenced from Zhang et al. (9). Defatted corn bran was added to water at the ratio of 1:20 (g/ml). Alkaline protease (0.74%) was added and the reaction mixture was incubated at 55°C and pH 10 for 4 h. Then it was stopped by boiling at 100°C for 15 min, and when cooled to 65°C, 0.73% starch complex enzyme [amylase: glucoamylase = 1:1 (*m*/*m*)] was added, adjusted to pH 6.7 simultaneously, and left for enzymolysis for 2 h. The reaction was terminated in the same way as above. After the mixture was cooled, it was centrifugated at 4,000 rpm for 15 min, and the residue was washed with 95% ethanol four times and dried at 45°C for 6 h to obtain the IDF from corn bran.

### Preparation of IDF With Different Particle Sizes From Corn Bran

The ultrafine pulverizer **(**FDV, Youqi, Beijing, China) was used to crush the IDF from corn bran, and the powder was sieved with different mesh sieves from 200 to 400 mesh (conformed to the provisions of Chinese national standards, GB 5330-85). The size of the sieve pores were 75, 63, 53, 45, and 38 μm respectively. The powder of the control (CK) group was sieved with 60 mesh (the size of the sieve pore, 250 μm). The evaluation of the particle size was undertaken using a laser diffraction particle size analyzer (Bettersize 2000, Better, Dandong, China).

### Physicochemical Properties Determination

#### Water-Holding Capacity and Oil-Holding Capacity

The WHC was determined using the process suggested in López-Marcos et al. (19) with some modifications. IDF samples were mixed with distilled water to make a solution that had a concentration of 5 %. The suspended matter was deposited and kept at 25°C for 4 h. Then it was centrifugated at 4,000 rpm for 15 min, and the sample was weighed. It was calculated using Equation (1):


(1) $$\begin{array}{l} WHC/\left(\text{g}/\text{g}\right)=\frac{W_{\text{w}}-W_{s}}{W_{\text{s}}} \end{array}$$

The OHC was determined by consulting the process suggested in Zheng et al. (20) with some modifications. 2.0 g of IDF was mixed with 16 g soybean oil, and the mixture was incubated at 25°C for 4 h. Then it was centrifugated at 4,000 rpm for 15 min. The sediment was weighed and the OHC was calculated using Equation (2):


(2) $$\begin{array}{l} OHC/\left(\text{g/g}\right)=\frac{W\text{w}-Ws}{W\text{s}} \end{array}$$

where *W*w is the wet weight of the sample, and *W*s is the weight of the sample.

#### Water Swelling Capacity

The WSC was determined using the process suggested in Zheng and Li (21) 1.0 g of IDF was mixed with 10 ml distilled water, and hydrated for 18 h at 25°C. The WSC was calculated using eqn (3):


(3) $$\begin{array}{l} WSC/\left(\text{ml/g}\right)=\frac{V_{1}-V_{0}}{W\text{s}} \end{array}$$

where *V*_1_ is the hydrated volume, *V*_0_ is the initial volume, and *W*s is the weight of the sample.

#### Cholesterol Adsorption Capacity and Cholate Adsorption Capacity

To determine CAC and CLAC, we referenced Yang et al. (22) 1.0 g of sample was mixed with 30 ml of diluted yolk solution (egg yolk and water, nine times the volume of yolk, added and mixed well). To simulate the gastric environment, the pH was adjusted to 2.0. It was further adjusted to pH 7.0 for simulating the small intestine environment. The mixture was incubated at 120 rpm at 37°C for 3 h. It was then centrifugated at 4,000 rpm for 15 min. The absorbance of the supernatant was measured at 550 nm and calculated as eqn (4):


(4) $$\begin{array}{l} CAC/\left(\text{mg/g}\right)=\frac{N_{1}-N_{2}}{W\text{s}} \end{array}$$

where *N*_1_ is the mass of cholesterol before absorption, *N*_2_ is the mass of cholesterol after absorption, and *W*s is the weight of the sample.

About 200 mg of the sample was mixed with 20 mg sodium cholate or sodium taurocholate in 20 ml of buffer (pH 7.0), shaken well, and incubated at 120 rpm at 37°C for 2 h. It was then centrifugated at 4,000 rpm for 15 min, and the absorbance of the supernatant was measured by the furfural colorimetric method and calculated as eqn (5):


(5) $$\begin{array}{l} CLAC/\left(\text{mg/g}\right)=\frac{\left(C_{1}-C_{2}\right)\times 20}{W\text{s}} \end{array}$$

where *C*_1_ is the initial concentration of cholate, *C*_2_ is the terminal concentration of cholate, 20 is the volume of buffer, and *W*s is the weight of the sample.

### Structural Characterization of IDF

#### Scanning Electron Microscopy

The samples were analyzed under a SU8020 scanning electron microscope (Hitachi, Tokyo, Japan) at an accelerating potential of 5.0 kV.

#### X-Ray Diffraction

The XRD was detected by the D8 Advance diffractometer (Bruker, Karlsruhe, Germany) with Ni-filtered Cu-Kα radiation (40 kV, 30 mA). A 2θ range from 5° to 70° at a speed of 0.02° s^−1^ was used for the XRD.

#### Fourier Transform Infrared Spectroscopy

About 5 mg of IDF samples were mixed with 100 mg KBr and the FT-IR of the sample was analyzed under a Spectrum Two Fourier transform infrared spectrophotometer (Bruker, Karlsruhe Germany), at a range of 400–4,000 cm^−1^.

### *In vitro* Hypoglycemic Activity

#### Glucose Adsorption Capacity

About 1.0 g of the IDF samples were weighed and mixed with 100 ml of glucose standard solution (10, 20, 35, and 50 mmol/L). The mixture was incubated at 120 rpm at 37°C for 6 h. Then it was centrifugated at 4,000 rpm for 15 min. The content of glucose in the supernatant was measured according to the instructions in the kit, and calculated using the formula (6):


(6) $$\begin{array}{l} GAC/\left(\text{mmol/g}\right)=\frac{N_{i}-N_{e}}{W\text{s}} \end{array}$$

where *N*_*i*_ is the amount of initial glucose, *N*_*e*_ is the amount of glucose at the end of the reaction, and *W*s is the weight of the sample.

#### Glucose Dialysis Retardation Index

The method was referenced from Zhu et al. (23) with some modifications. About 0.5 g of the sample was mixed with 25 ml glucose solution (50 mmol/L) into a dialysis bag (8,000 MWCO, Solarbio Science and Techno-logy Co., Ltd, Beijing, China) with 200 ml of distilled water as the dialysis solution in a shaker (120 rpm), at 37°C. Then 1.0 ml of the dialysis solution was taken at 10, 30, 60, 90, 120, 150, and 180 min, and the glucose concentration was measured using the kit. The group without an IDF sample was treated as a control.

### Effect on Starch Digestion *in vitro*

It was determined using the previous method (23). A sample of 50 mg corn starch and 20 mg IDF was weighed and mixed with 10 ml buffer (pH5.2) and incubated at 37°C for 10 min. About 4 mg α-amylase (50 U/mg) and 1 ml glucosidase (0.4 U/ml) were added to the mixture in a shaker (120 rpm). The reaction time was set at 10, 20, 30, 60, 90, and 120 min respectively, then boiled to inactive the enzyme. After cooling, it was centrifuged at 4,000 rpm for 15 min. The concentration of glucose in the supernatant was measured using the kit. The group without an IDF sample was treated as a control.

### Effect on α-Glucosidase Activity Inhibition

The method was adapted from Chen et al. (24) with some modifications. About 1.0 ml of PBS buffer solution (pH 6.8) was taken in a blank control tube, and 0.5 ml of α-glucosidase (0.4 U/ml) was added to the blank tube. About 20 mg of sample and 0.5 ml α-glucosidase were added to the sample tube. And 20 mg of sample was added to the sample control tube. Each tube was supplemented with PBS buffer solution until the solution volume was 1.5 ml at 37°C for 10 min. Then 1.0 ml of PNPG solution (10 mmol/L) was added to each tube, and 5 ml of Na_2_CO_3_ (0.2 mol/L) was added immediately after 30 min. The absorbance was measured at 405 nm after mixing. The inhibition rate of the sample to α-glucosidase was calculated as follows:


(7) $$\begin{array}{l} \text{Inhibition activity }\left(\%\right)=\left(1-\frac{A_{3}-A_{4}}{A_{2}-A_{1}}\right)\times 100\% \end{array}$$

Where A_1_, A_2_, A_3_, and A_4_ are the absorbance values of blank control tube, blank tube, sample tube, and sample control tube at 405 nm respectively.

### Statistical Analysis

All the data were measured three times and showed as “mean ± standard error.” The data were analyzed by Duncan's test and ANOVA using SPSS 20.0 software. A probability level of 5% (*p* < 0.05) was regarded as statistically significant. The Pearson correlation between physical (mesh, D50, D_[4,3]_, D_[3,2]_, specific surface area and the content of Cellulose, Hemicellulose, Lignin and the functional properties of (WHC, OHC, WSC, CAC(pH2 and pH7), CLAC (SC and STC), GAC(50 mmol), GDRI, Starch digestion inhibition, and α-glucosidase activity inhibition) were calculated.

## Results

### Particle Size Distribution of IDF From Corn Bran

**Figure 1:**
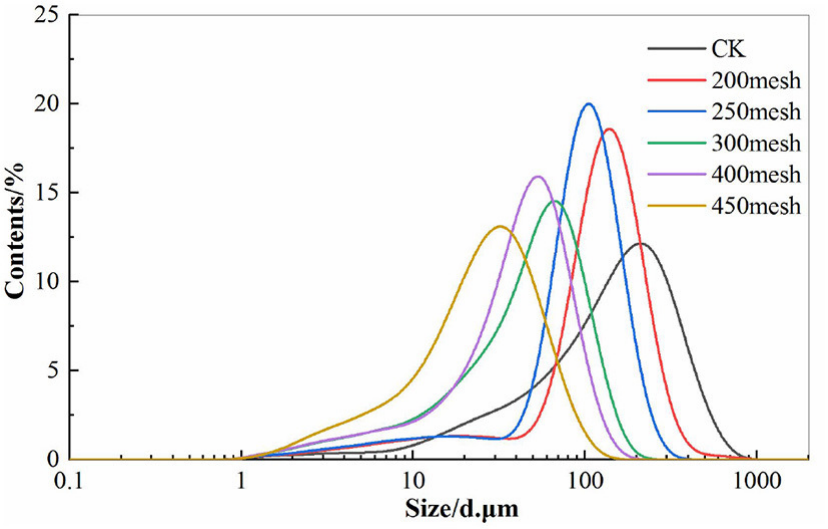
Particle size distribution of IDF from corn bran. CK: IDF from corn bran (about 60 mesh), the rest of the samples were crushed by ultrafine pulverizer and sieved with different mesh sieves from 200 to 400 mesh, Subsequent test samples were processed according to this method.

The particle size distribution peaks of powder shifted to the left after the corn bran IDF ultrafine pulverized and sieved with different mesh. Compared with CK, and the peak height increased, the span narrowed, indicating that the particle size distribution of IDF after ultrafine pulverization was relatively concentrated and uniform (Figure 1). On the other hand, the median diameter (D50), volume average diameter (D_[4,3]_), and area average diameter (D_[3,2]_) of corn bran IDF after ultrafine pulverization were significantly decreased (*p* < 0.05). The D98 value of corn bran IDF sieved with 200–400 mesh, compared with the CK group, was decreased by 38.38, 57.06, 72.86, 77.78, and 83.44%, respectively. The specific surface area of IDF increased significantly (*p* < 0.05) by 14.58, 31.25, 120.83, 131.25, and 225.00%, respectively (Table 1). The study found that the change of the specific surface area affected the adsorption capacity of the powder to the solvent, and it had great potential as a food additive or active ingredient (15).

**Table 1:** Particle size distribution of IDF from corn bran.

**Mesh**	**D50 (μm)**	**D90 (μm)**	**D98 (μm)**	**D_**[4, 3]**_ (μm)**	**D_**[3, 2]**_ (μm)**	**Specific surface area (m^**2**^/kg)**
CK	134.1 ± 0.20^a^	314.4 ± 1.10^a^	454.6 ± 2.30^a^	154.5 ± 0.32^a^	40.12 ± 0.08^a^	0.048 ± 0.004^f^
200	112.2 ± 0.20^b^	200.3 ± 1.20^b^	280.1 ± 1.60^b^	117.5 ± 0.27^b^	34.63 ± 0.09^b^	0.055 ± 0.003^e^
250	86.26 ± 0.14^c^	147.4 ± 1.30^c^	195.2 ± 1.10^c^	88.05 ± 0.18^c^	30.46 ± 0.08^c^	0.063 ± 0.004^d^
300	45.00 ± 0.09^d^	90.98 ± 0.67^d^	123.4 ± 0.80^d^	47.99 ± 0.10^d^	18.10 ± 0.07^d^	0.106 ± 0.008^c^
350	38.84 ± 0.12^e^	74.54 ± 0.53^e^	101.0 ± 0.65^e^	40.95 ± 0.09^e^	17.30 ± 0.07^e^	0.111 ± 0.009^b^
400	23.06 ± 0.15^f^	51.87 ± 0.45^f^	75.28 ± 0.49^f^	26.47 ± 0.11^f^	12.37 ± 0.05^f^	0.156 ± 0.007^a^

*Using distilled water as the medium, the sample was uniformly dispersed by ultrasonic assistance. Different letters of the same index showed a significant difference at p < 0.05*.

### Chemical Compositions and Physicochemical Properties of IDF From Corn Bran

**Figure 2:**
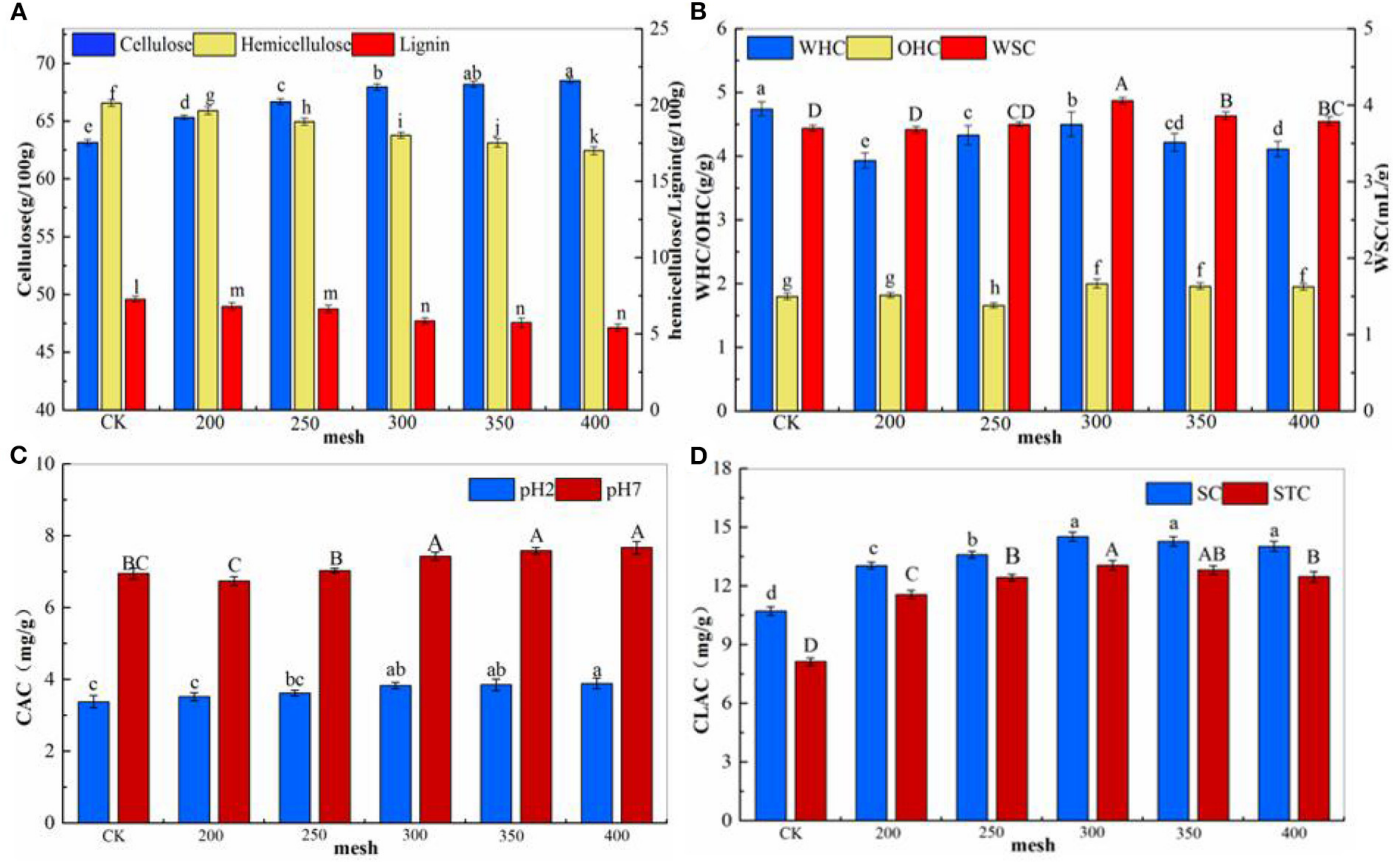
Chemical compositions and physicochemical properties of different particle size IDF from corn bran. **(A)** Contents of cellulose, hemicellulose, and lignin, **(B)** WHC, OHC, and WSC of different particle size IDF from corn bran, **(C)** the CAC of different particle size IDF from corn bran, and **(D)** the CLAC of different particle size IDF from corn bran. Different letters of the same index showed a significant difference at *p* < 0.05.

IDF includes cellulose, lignin, and some hemicelluloses (25). The contents of cellulose were significantly increased and the content of hemicellulose and lignin was decreased, compared with CK when the sieving mesh was >200 (*p* < 0.05), (Figure 2A). The WHC of corn bran IDF was significantly lower than CK after ultrafine pulverization (*p* < 0.05), and WSC and OHC were significantly increased when the mesh was >250 mesh (*p* < 0.05) (Figure 2B). Among them, the WHC of 300 mesh IDF decreased by 5.06%, the WSC increased by 9.73% and the OHC increased by 10% compared with the CK group. Powders with good water-holding and water-swelling properties can increase defecation and reduce the risk of bowel cancer and other diseases (2, 26), while powders with good oil-holding properties can absorb oil, thereby, the intake of oil in the body is greatly reduced (27). The characteristic of CAC can reduce the loss of fat, which is more conducive to the absorption of lipids in the intestines so that the content of cholesterol in the serum is reduced, resulting in the lowering of blood lipids (28). The CAC *in vitro* test can also evaluate effectively the adsorption capacity of DF in simulated gastric (pH 2) and intestinal (pH 7) environments. The CAC was higher under neutral than acidic conditions, and the CAC of corn bran IDF was significantly increased (*p* < 0.05) when the particle size was larger than 300 mesh (Figure 2C). The CLAC of the ultrafine pulverized corn bran IDF was significantly increased (*p* < 0.05) (Figure 2D), indicating that the processing had a positive effect on CLAC, and the adsorption effect of sodium cholate (SC) was better than that of sodium taurocholate (STC). These findings were consistent with other studies such as Yu et al. (29), where superfine grinding modified carrot pomace dietary fiber (5), and Wang (2), demonstrated that using superfine grinding technology could improve the performance of pea DF (2).

### SEM of Different Particle Size IDF From Corn Bran

**Figure 3:**
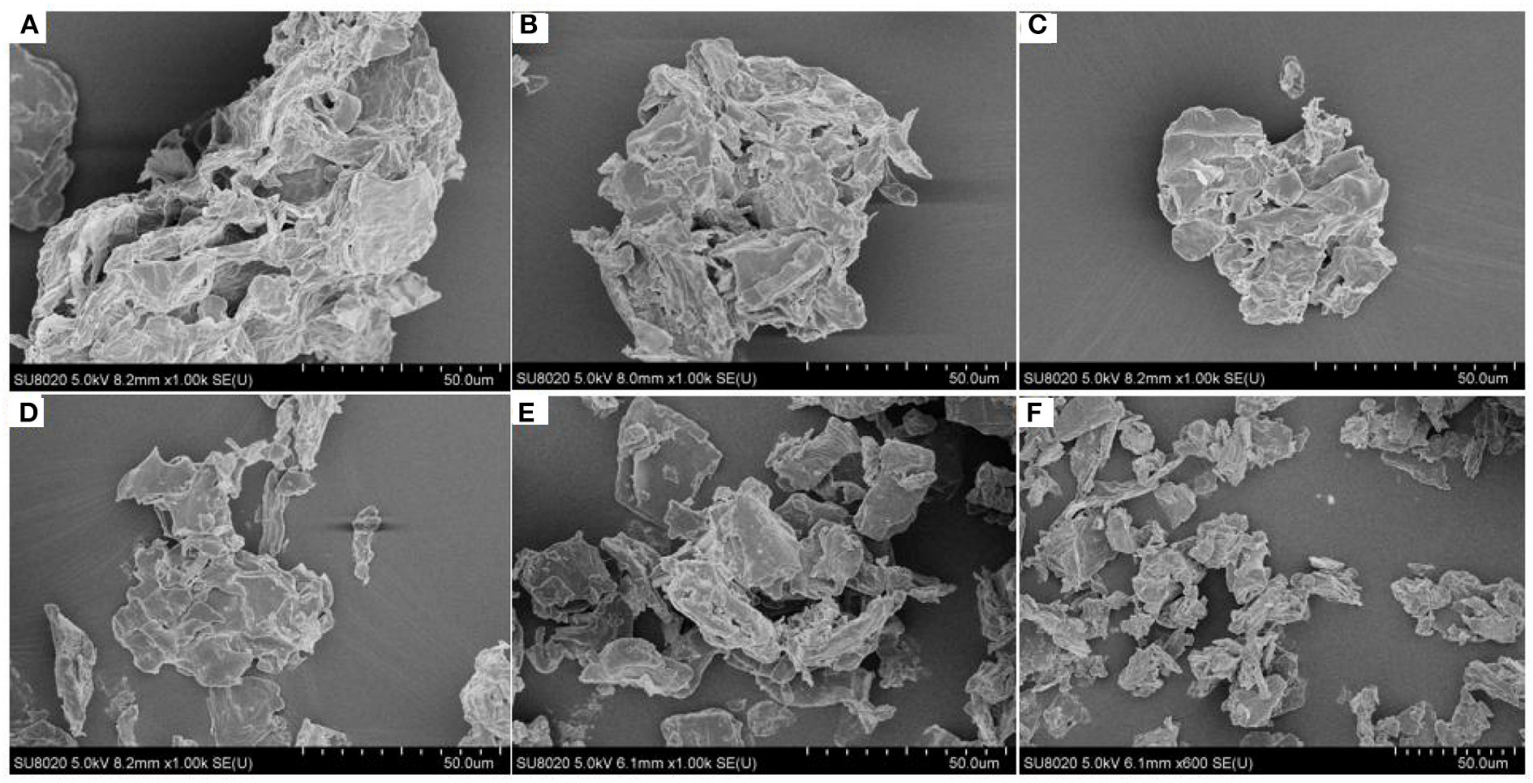
The SEM of CK **(A)**, 200 mesh **(B)**, 250 mesh **(C)**, 300 mesh **(D)**, 350 mesh **(E)**, and 400 mesh **(F)** at magnification 1000×.

Under the same magnification of scanning electron microscope, the CK group had a larger particle size, more obvious surface folds, irregular edge, and non-uniform porous structure (Figure 3). Due to the ultrafine grinding, part of the structure of the fiber had been destroyed to a certain extent, and its particles have changed significantly. The sample groups had smaller particle sizes and looser structures compared with the CK group. After ultrafine grinding, the particle size was decreased, especially when the D50 of the IDF sample was <40 μm, the degree of granulation was more obvious, and the structure was slightly looser. The change of the IDF structure was more favorable for dissolution and improved the property of the IDF (30).

### XRD of Different Particle Size IDF From Corn Bran

**Figure 4:**
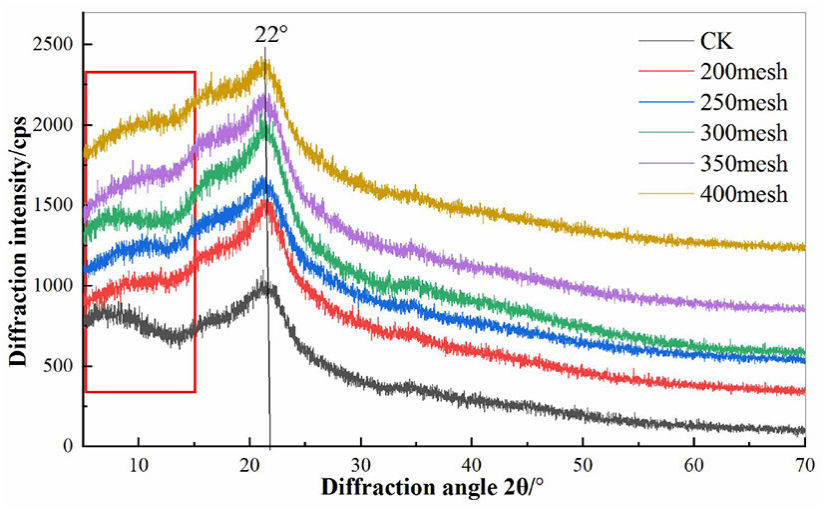
The X-ray diffraction (XRD) of different particle size IDF from corn bran.

The corn bran IDF has an obvious diffraction peak around 22°, and a weak diffraction peak around 5°~15° (Figure 4). The overall diffraction intensity of the corn bran IDF was increased with the decrease of particle size, which indicated that the relative crystallinity of IDF increased significantly. The diffraction peak at about 20°~23° represents the cellulose type I with a double helix (31). The diffraction peak at about 10° represents the type II structure of cellulose. When the diffraction angle is 22°, the strong diffraction peak is the characteristic peak of the cellulose crystal structure (32). The results also showed that the cellulose content in the IDF was increased with the decrease in particle size.

### FT-IR of Different Particle Size IDF From Corn Bran

**Figure 5:**
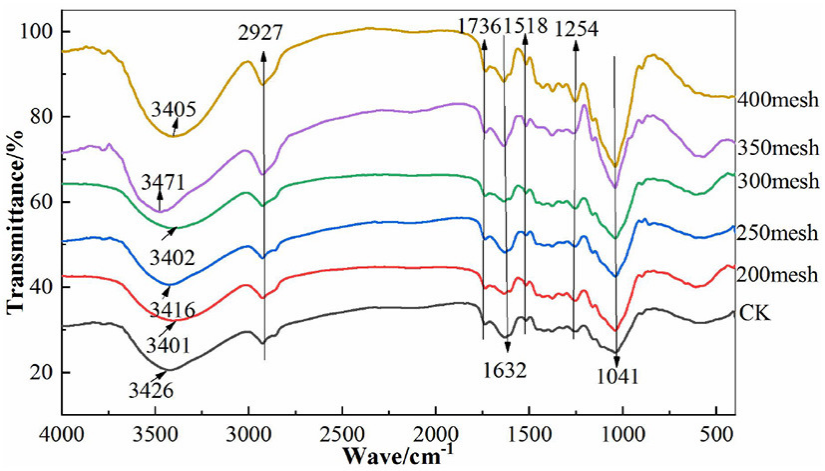
The FTIR spectrogram of different particle size IDF from corn bran.

The peak positions of the characteristic peaks of corn bran IDF with different particle sizes were very similar (Figure 5), indicating that the particle size had not changed the characteristic absorption of IDF in the infrared spectrum. IDFs with different particle sizes all showed a strong and smooth absorption peak around 3,500 cm^−1^, which was generated by the O-H stretching vibration of cellulose or hemicellulose (33). The absorption peak at 2,927 cm^−1^ represented the C-H contraction vibrations of some methylene groups, representing the cellulosic typical structure (34). The bands at 1,736 and 1,632 cm^−1^ were generated by the C=O asymmetric stretching vibration of the esterification, indicating that the corn bran IDF contained -CHO or -COOH (35). The absorption peak at 1,514 cm^−1^ represented the stretching and vibration of carboxyl groups (36). The peak at 1,044 and 1,254 cm^−1^ was due to the sugar rings C-O and C-O-H formed by the stretching vibration of C-O in cellulose and hemicellulose, as well as the variable angle vibration of O-H. The strength of the absorption peak increased with the decrease in particle size, indicating that the ultrafine grinding broke down the intermolecular forces and exposed more -OH and C-O groups. It has been found that this was also an important adsorption site for heavy metals (37, 38). 500–1,000 cm^−1^ was mainly the peak of carbohydrate absorption (23).

### *In vitro* Hypoglycemic Activity of Different Particle Size IDF From Corn Bran

**Figure 6:**
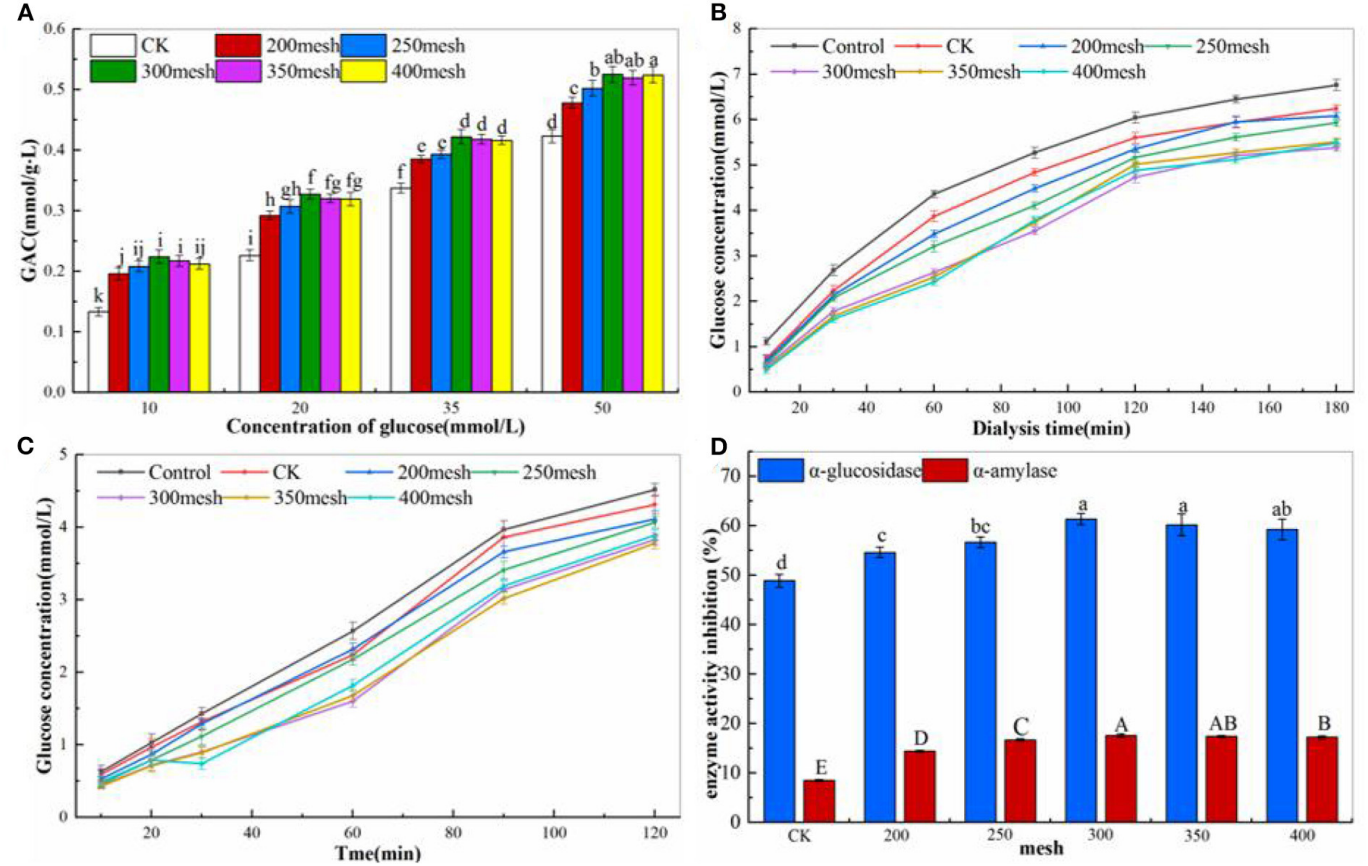
*In vitro* hypoglycemic properties of different particle size IDF from corn bran. **(A)** The GAC of IDF. **(B)** Glucose diffusion inhibition of IDF. **(C)** Effect on starch digestion of IDF. **(D)** α-glucosidase and α-amylase inhibition of IDF. Different letters of the same index showed significant difference at *p* < 0.05.

Some studies have shown that IDF has hypoglycemic properties. The IDF has a great glucose absorption capacity. With the increase in glucose concentration (10–50 mmol/L), the adsorption characteristics of IDF gradually increased (Figure 6A). The maximum GAC of the CK group was 0.423 ± 0.100 mmol/g, while that of 300-mesh corn bran IDF was 0.525 ± 0.013 mmol/g, an increase of 24.11%. It also showed that the GAC of IDF, with the decrease in particle size, increased significantly (*p* < 0.05) at any glucose concentration, indicating that ultrafine grinding had a positive effect on GAC. The glucose in the dialysate was enhanced constantly with time (Figure 6B). Compared with the control group, the addition of IDF reduced the diffusion, and the glucose in the dialysate was significantly decreased when the sieving was >250 mesh. The concentration of glucose in dialysate decreased from 1.27 to 1.38 mmol/l when the dialysis time was 180 min. The effect of IDF on starch digestion is shown in Figure 6C. The IDF reduced the concentration of glucose, and it also decreased significantly when the sieving was >250 mesh. The concentration of glucose decreased from 0.63 to 0.74 mmol/l when the digestion time was 120 min. At last, α-glucosidase and α-amylase inhibitors were widely used to regulate postprandial blood glucose in patients through their competitive inhibition action (39). The inhibitory activity of α-glucosidase and α-amylase was increased significantly with the decrease in particle size (*p* < 0.05), and the inhibitory activity of α-glucosidase was stronger than α-amylase (Figure 6D). When the IDF was 300 mesh, the inhibitory rate of α-glucosidase was 61.34 ± 1.12%, and the inhibitory rate of α-amylase was 17.58 ± 0.33%, exhibiting an increase of by 25.54 and 106.83%, respectively.

### Pearson Correlation Between Physical and Functional Properties

**Figure 7:**
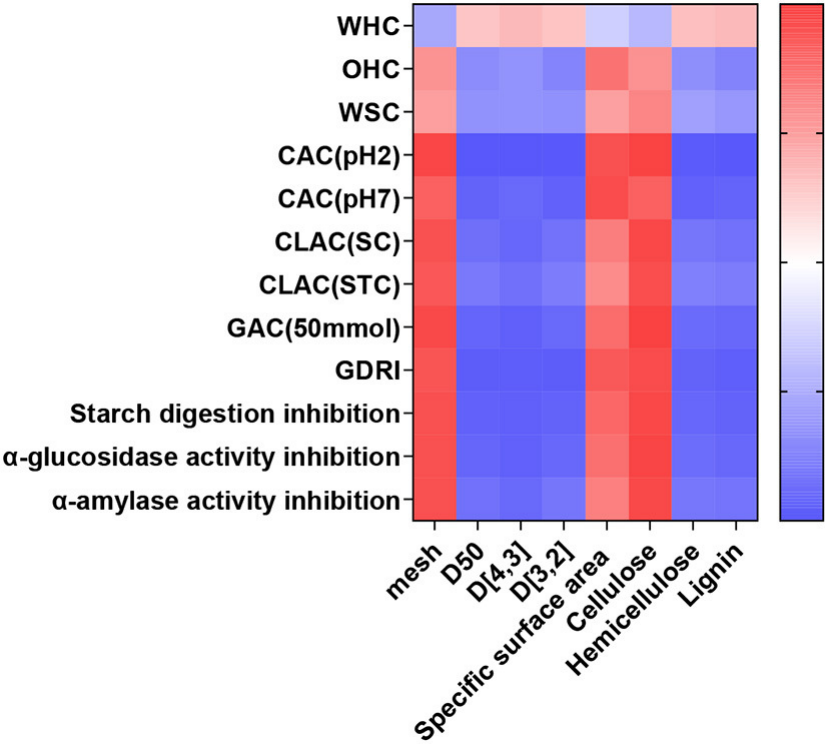
Correlation between physical and functional properties of IDF from corn bran.

The Pearson correlation between physical and functional properties was calculated and the results are shown in Figure 7. The correlation between mesh size and CAC (pH2), and GAC (50 mmol/L) yielded high positive r values of 0.966 and 0.952, respectively. The correlation between D50 and CAC (pH2), and GDRI generated high negative r values of 0.996 and 0.965, respectively. The correlation between D_[4,3]_ and CAC (pH2), GAC (50 mmol/L), GDRI, and starch digestion inhibition were all high negative r values >0.95. The correlation between D_[3,2]_ and CAC (pH2), and GDRI yielded high negative r values of 0.992 and 0.966, respectively. The correlation between the content of cellulose and CAC (pH2), CLAL (SC), GAC (50 mmol/L), starch digestion inhibition, α-glucosidase activity inhibition, and α-amylase activity inhibition had high positive r values >0.95. The correlation between the content of hemicellulose and CAC (pH2) generated high negative r values of 0.981. Correlations between the content of lignin and CAC (pH2), and GDRI yielded high negative r values of 0.990 and 0.955 respectively.

## Discussion

Studies have shown that particle size can directly measure the effect of ultrafine grinding (2). In this paper, the particle size distribution of corn bran IDF was relatively concentrated after ultrafine grinding. The particle size was also uniform (Figure 1), and the D50, D_[4,3]_, and D_[3,2]_ were all decreased significantly (*p* < 0.05), but the specific surface area increased significantly (*p* < 0.05). These illuminated the shear, the impact. The impact and other forces generated in the process of airflow ultrafine pulverization could reduce the particle size of the powder effectively. However, it is possible that the ultrafine pulverization destroyed the structure of fibers simultaneously, leading to the redistribution of fiber components. Cellulose content was significantly increased and the content of hemicellulose and lignin was decreased, compared with CK (*p* < 0.05) (Figure 2A). The result was consistent with Li et al., who demonstrated that under various strong forces of ultrafine pulverization, some insoluble hemicelluloses such as arabinoxylan and insoluble pectin compounds will be melted or partially broken, and converted into water-soluble polymer components. In our study, the processing altered the functional properties of the fibers and improved the physicochemical properties of DF effectively (29). There was a certain relationship between the WHC, OHC, and WSC of DF and the spatial structure of the network fiber.

According to the experimental data, the WHC was lower than the CK group. It could be that the structure of corn bran IDF was destroyed and the contact area or hydrophilic groups in dietary fibers was reduced, which weakened its binding ability to water. Previous studies have demonstrated that good WSC and OHC of DF can effectively prevent and treat obesity. In this paper, the WSC and OHC increased significantly (*p* < 0.05). Those results were possible because surface properties were changed, exposing lots of hydrogen bonds and dipole forms, thereby enhancing the WSC of the ultrafine powder. Additionally, the specific surface area increased, which may have promoted the WSC. The decrease in particle size also resulted in more exposure to some lipophilic groups, which enhanced the OHC of the ultrafine powder. In addition, the reduction of the particle size of IDF after ultrafine grinding impacted the binding ability to cholesterol. It is generally believed that the main mechanism of DF to reduce blood lipid and cholesterol is to absorb fat, increase the amount of fat excretion in feces, bind bile acid, and promote the conversion of cholesterol into bile acid. Several studies have shown that cellulose is more hydrophobic than hemicellulose in the major constituents of IDF (40). The higher content of cellulose in IDF with smaller particle sizes may be the reason for its higher adsorption properties (22).

The surface of corn bran IDF was porous and uneven. The ultrafine grinding resulted in severe tearing, which lead the particle to become smaller and the structure looser which increased the surface area of IDF compared with the CK group. Due to the strong external force during the ultrafine grinding, part of the fiber's structure was destroyed to a certain extent and the particles changed noticeably resulting in the functional properties of the fiber being affected greatly (41). It can be seen from the X-RAD that the corn bran IDF has characteristic structure peaks of a typical cellulose type I structure with a double helix, and cellulose type II structure respectively. However, the crystallinity of IDF increased slightly with the decrease in particle size. It may be due to the decrease in hemicellulose and lignin content in IDF by ultrafine grinding, resulting in the destruction of the amorphous region. According to the infrared spectra data, the characteristic absorption peaks of IDF with different particle sizes were approximately the same (Figure 5), having the typical structure of cellulose and also containing -CHO or -COOH groups. A change in the hydrogen bond might have loosened the fiber structure (42). During the ultrafine grinding, glycosidic bonds were broken, which disintegrated the IDF structure, exposing more -OH and C-O groups.

The specific surface area of corn bran IDF increased significantly with decreasing particle size (*p* < 0.05), exposing more functional groups, such as -OH and -COOH, so the van der Waals and hydrogen bonding forces could effectively adsorb glucose molecules (43). At the same time, the effect of IDF of different particle sizes on glucose diffusion was studied *in vitro*. The main mechanism is that IDF samples can change the viscosity of the solution and have the characteristic of glucose adsorption, which can reduce the diffusion rate and glucose content. The effect on starch digestion showed that IDF from corn bran could inhibit starch digestion, and the inhibition mechanism may be due to the direct action of IDF surface enzyme inhibitors on the enzyme. The decrease in particle size and the increase in specific surface areas exposed more binding sites to the enzyme and made it inactive. The results showed that the inhibitory activity of IDF on α-glucosidase was stronger than α-amylase, which may be related to the binding of IDF to polyphenols. This could delay the absorption of carbohydrates and control postprandial blood glucose more effectively (44).

There was a certain correlation between the particle size, composition of IDF, and its functional properties. The particle size was highly negatively correlated with CAC (PH2), GAC (50 mmol/L), GDRI, and starch digestion inhibition (2, 5). The cellulose content of IDF was positively correlated with the functional properties of IDF except for WHC, while the content of hemicellulose and lignin was negatively correlated with the functional properties of IDF except for WHC. There was a high positive correlation between cellulose and CAC (pH2), CLAC (SC), GAC (50 mmol/L), starch digit inhibition, α-glucosidase activity inhibition, and α-amylase activity inhibition. A possible reason is that the porous structure of the fiber itself and the chemical groups on the surface of the molecule was more favorable for improving the properties of IDF.

## Conclusion

To explore the effect of particle size on the IDF and to expand its application channels, in this study, the corn bran IDF with different particle sizes was compared and analyzed for its physicochemical properties, microstructure, and *in vitro* hypoglycemic activity. The results showed that, for most functional properties, 300 mesh (D50 value of about 45 μm) had the best quality, such as OHC, WSC, CAC, and CLAC. The structure of IDF was observed by SEM, X-ray, and FT-IR. It was found that the surface was porous and uneven and the structure of the fiber was destroyed by ultrafine grinding. The particle became smaller and the crystallinity of IDF increased slightly. More -OH and C-O groups were exposed, which could adsorb glucose effectively. At the same time, the IDF surface enzyme inhibitors effectively reduced the degree of starch digestion and controlled postprandial blood glucose levels. In addition, it was found by correlation analysis that the particle size was highly negatively correlated with some functional properties of IDF, and the cellulose content was positively correlated with the functional properties of IDF except WHC. To sum up, reducing particle size was suitable for the development of IDF in high value-added products.

## Data Availability Statement

The original contributions presented in the study are included in the article/supplementary material, further inquiries can be directed to the corresponding authors.

## Author Contributions

XZ and XL were responsible for the design and management of the entire experiment. JW provided a part of the test route. FZ performed experimental methods and collected the data. CJ and RW analyzed the data and wrote the manuscript. All authors have read and agreed to the manuscript for publication.

## Funding

This work was supported by cooperation research on the Key Technology of Corn Functional Sugar Production (2019ZX06B02), Heilongjiang Key R&D Program Guidance Project (GZ20210097), Heilongjiang One Billion in Major Engineering and Technology Sub-project (2021ZX12B09-4), and Talent Foundation of the Central Government Supporting Local Universities (2020GSP08).

## Conflict of Interest

The authors declare that the research was conducted in the absence of any commercial or financial relationships that could be construed as a potential conflict of interest.

## Publisher's Note

All claims expressed in this article are solely those of the authors and do not necessarily represent those of their affiliated organizations, or those of the publisher, the editors and the reviewers. Any product that may be evaluated in this article, or claim that may be made by its manufacturer, is not guaranteed or endorsed by the publisher.
